# Nationwide projections of ischemic stroke with large vessel occlusion of the anterior circulation by 2050: Dijon Stroke Registry

**DOI:** 10.3389/fpubh.2023.1142134

**Published:** 2023-05-25

**Authors:** Gauthier Duloquin, Yannick Béjot

**Affiliations:** Dijon Stroke Registry, Department of Neurology, University Hospital of Dijon, Pathophysiology and Epidemiology of Cardio-cerebrovascular Disease (PEC2), University of Burgundy, Dijon, France

**Keywords:** ischemic stroke, registries, epidemiology, large vessel occlusion, population-based studies

## Abstract

**Introduction:**

Data on the epidemiology of ischemic stroke (IS) with large vessel occlusion (LVO) are scarce although there is a need to better assess future demands for dedicated facilities in an aging population. This study aimed to estimate the number of expected cases of IS with LVO of the anterior circulation in the French population by 2050.

**Methods:**

IS were retrieved from the population-based registry of Dijon, France (2013–2017). Patients with LVO were identified and age-and sex-standardized incidence rates were calculated to estimate the number of expected cases in the whole French population by 2050 according three scenarios: stable incidence; a decrease in incidence rates of 0.5%/year in people >65 years old; a decrease in incidence rates of 0.5%/year in overall population.

**Results:**

1,067 cases of IS with LVO were recorded in Dijon over the study period, corresponding to crude incidence rate of 22/100,000/year (95% CI: 18–25). By 2050, the number of cases is expected to increase by 51 to 81% according to the various scenarios, to reach between 22,457 cases (95% CI: 10,839 – 43,639) and 26,763 cases (95% CI: 12,918 – 52,008) annually. This increase will be mainly driven by patients >80  years old, with a rise of cases between +103% and +42% in this age group. The proportion of patients >80 years old among overall IS with LVO will increase from 43 to 57% approximately.

**Conclusion:**

The expected massive increase in IS with LVO highlights the need for a rapid action to cover stroke care requirements.

## 1. Introduction

With the advent of mechanical thrombectomy (MT) as acute treatment of ischemic stroke (IS) due to large vessel occlusion (LVO) of the anterior circulation, patients’ outcome dramatically improved (1). Although MT is now considered as the standard of care of IS with LVO therapy, in addition with intravenous thrombolysis when indicated (2), it can be performed only in centres with interventional neuroradiology facilities, the number of which is limited in a vast majority of countries. This shortage in specialised medical resources poses a number of challenges for pre-hospital triage of patients suspected of suffering an IS, and the referral to MT-capable centres. Because of the ongoing aging population, an increase in the annual total number of strokes has been observed over the past decades, and this trend will dramatically magnify in coming years (3–5). These epidemiological perspectives represent a threat to the sustainability of health systems. In particular, there is an urgent need to plan for the future resources required for the acute management of IS patients with LVO.

Therefore, the aim of this study was to estimate the number of expected cases of IS with LVO of the anterior circulation in the French population by 2050.

## 2. Materials and methods

### 2.1. Study population and calculation of incidence rates

All cases of IS were retrieved from the Dijon Stroke Registry (6, 7), an ongoing prospective population-based study that complies with the criteria for conducting “ideal” incidence stroke studies, and the guidelines for the reporting of incidence and prevalence studies in neuroepidemiology according to Standard of Reporting of Neurological Disorders (STROND). The methodology of the Dijon Stroke Registry has been described extensively elsewhere (6, 7). Briefly, case-collection relies on multiple overlapping sources of information to identify first-ever and recurrent hospitalized and not hospitalized cases of stroke among residents of the city of Dijon France (currently 156,000 inhabitants). For this study, analyses were restricted to IS patients registered between 1st January 2013 and 31st December 2017.

### 2.2. Assessment of large vessel occlusion

To assess the presence of a LVO in IS patients, all cervical and intracranial arterial imaging exams were systematically reviewed by stroke-trained investigators. A LVO of the anterior circulation was defined as an occlusion site affecting the terminal intracranial internal carotid artery, M1 and M2 segments of the middle cerebral artery (including tandem occlusions), or A1 and A2 segment of the anterior cerebral artery. Patients with isolated extracranial internal carotid artery occlusion were not included in this group since the benefit of MT in them has still to be demonstrated. In addition, LVO was considered only if it was responsible for the acute IS.

### 2.3. Statistical analyses

Annual incidence rates of IS with a proximal occlusion of the anterior circulation were calculated according to age groups, and sex, and were expressed per 100,000 people with 95% CIs assuming a Poisson distribution as previously reported (6). Denominators concerning the population of Dijon were based on census data provided by the French National Institute of Statistics. The estimation of the number of cases of IS with LVO of the anterior circulation in France was obtained by standardization to age-and sex-specific incidence found in the Dijon Stroke Registry over the period 2013–2017 (6). Expected number of cases until 2050 were assessed according to three different scenarios: stable age-and sex-specific incidence rates, a decrease in incidence rates of 0.5%/year in people over 65 years old, a decrease in incidence rates of 0.5%/year in overall population. The number of French inhabitants by 2050 was obtained by data from the Institut National de la Statistique et des Etudes Economiques (8). Statistical analysis was performed with STATA 13 software (StataCorp LP, College Station, TX, United States).

### 2.4. Ethics statement

The Dijon Stroke Registry was approved by following national ethics boards: the Comité d’Evaluation des Registres (French National Committee of Registers), Santé Publique France (French Institute for Public Health Surveillance), and the Commission Nationale Informatique et Liberté (French data protection authority). In accordance with the French legislation, boards waived the need for written patient consent.

## 3. Results

From 2013 to 2017, 1,060 cases of acute IS were recorded (mean age: 76.0 ± 15.8 years, 53.9% women). Information about arterial imaging was available in 971 (91.6%) patients. A LVO of the anterior circulation was observed in 167 patients (17.2%). Corresponding crude incidence rate was 22/100,000/year (95% CI: 18–25). Based on the age and sex-standardized incidence rates, we estimated that the number of IS with LVO in France in 2015 was 14,824 cases per year (95% CI: 6,540 – 31,371), including 8,190 cases in women (95% CI: 3,992 – 16,351) and 6,634 cases in men (95% CI: 2,548–15,020) (Table 1).

**Table 1:** Annual incidence rates of ischemic stroke with a proximal occlusion of the anterior circulation in Dijon, France, from 2013 to 2017 (expressed as n/100,000/year) and projection of absolute number of cases in the French population in 2015.

	Annual incidence rate of IS With proximal occlusion of the anterior circulation		French population in 2015	Number of cases estimated in France in 2015	
	Rate	(95% CI)		*N*	(95% CI)
Men					
<35	0.5	(0.0–2.9)	14,306,323	74	(2–415)
35–40	9.3	(1.1–33.4)	2,005,652	186	(22–671)
40–45	4.5	(0.1–24.9)	2,270,621	101	(3–564)
45–50	10.1	(1.2–36.6)	2,223,203	225	(27–814)
50–55	10.4	(1.3–37.6)	2,192,525	228	(27–824)
55–60	31.3	(11.5–68.1)	2,049,745	641	(235–1,396)
60–65	15.6	(3.2–45.6)	1,952,952	305	(63–889)
65–70	44.9	(18.0–92.4)	1,754,830	787	(317–1,622)
70–75	85.1	(38.9–161.6)	1,127,213	960	(439–1,821)
75–80	100.7	(46.0–191.1)	959,177	966	(442–1,833)
80–85	120.0	(54.9–227.9)	739,964	888	(406–1,686)
85–90	127.7	(46.9–278.0)	417,507	533	(196–1,161)
>90	395.4	(197.4–707.5)	187,195	740	(369–1,324)
Total	18.7	(14.5–23.7)	32,186,907	6,634	(2,548–15,020)
Women					
<35	0.5	(0.0–2.9)	14,021,501	72	(2–401)
35–40	4.7	(0.1–26.3)	2,053,333	97	(2–540)
40–45	9.6	(1.2–34.7)	2,296,368	220	(27–796)
45–50	19.1	(5.2–48.9)	2,279,838	435	(119–1,114)
50–55	13.5	(2.8–39.5)	2,278,487	308	(63–900)
55–60	8.8	(1.1–31.7)	2,179,570	191	(23–690)
60–65	8.6	(1.0–31.0)	2,120,386	182	(22–656)
65–70	19.4	(5.3–49.6)	1,942,726	376	(103–963)
70–75	42.6	(15.6–92.7)	1,312,221	559	(205–1,216)
75–80	122.3	(68.5–201.7)	1,253,150	1,533	(858–2,528)
80–85	102.0	(55.8–171.2)	1,149,794	1,173	(641–1,968)
85–90	145.7	(86.3–230.2)	819,152	1,193	(707–1,886)
>90	348.2	(229.4–506.6)	531,528	1,851	(1,220–2,693)
Total	24.3		34,238,054	8,190	(3,992–16,531)
Men and women					
<35	0.5	(0.0–1.9)	28,327,824	146	(4–816)
35	7.0	(1.4–20.5)	4,058,985	283	(25–1,210)
40	6.9	(1.4–20.3)	4,566,989	321	(29–1,360)
45	14.7	(5.4–32.1)	4,503,041	660	(146–1,928)
50	12.1	(3.9–28.2)	4,471,012	536	(91–1,724)
55	09.0	(8.2–37.5)	4,229,315	832	(258–2,086)
60	11.7	(3.8–27.4)	4,073,338	487	(85–1,546)
65	30.3	(15.1–54.3)	3,697,556	1,163	(419–2,585)
70	60.8	(34.0–100.3)	2,439,434	1,519	(644–3,037)
75	113.2	(72.5–168.4)	2,212,327	2,499	(1,299–4,361)
80	108.4	(68.7–162.7)	1,889,758	2,061	(1,048–3,655)
85	140.7	(90.2–209.4)	1,236,659	1,726	(903–3,046)
>90	360.6	(255.2–495.0)	718,723	2,591	(1,589–4,017)
Total	21.7	(18.5–25.2)	66,424,961	14,824	(6,540–31,371)

IS, Ischemic Stroke.

### 3.1. Projection of the total number of is with LVO

**Figure 1:**
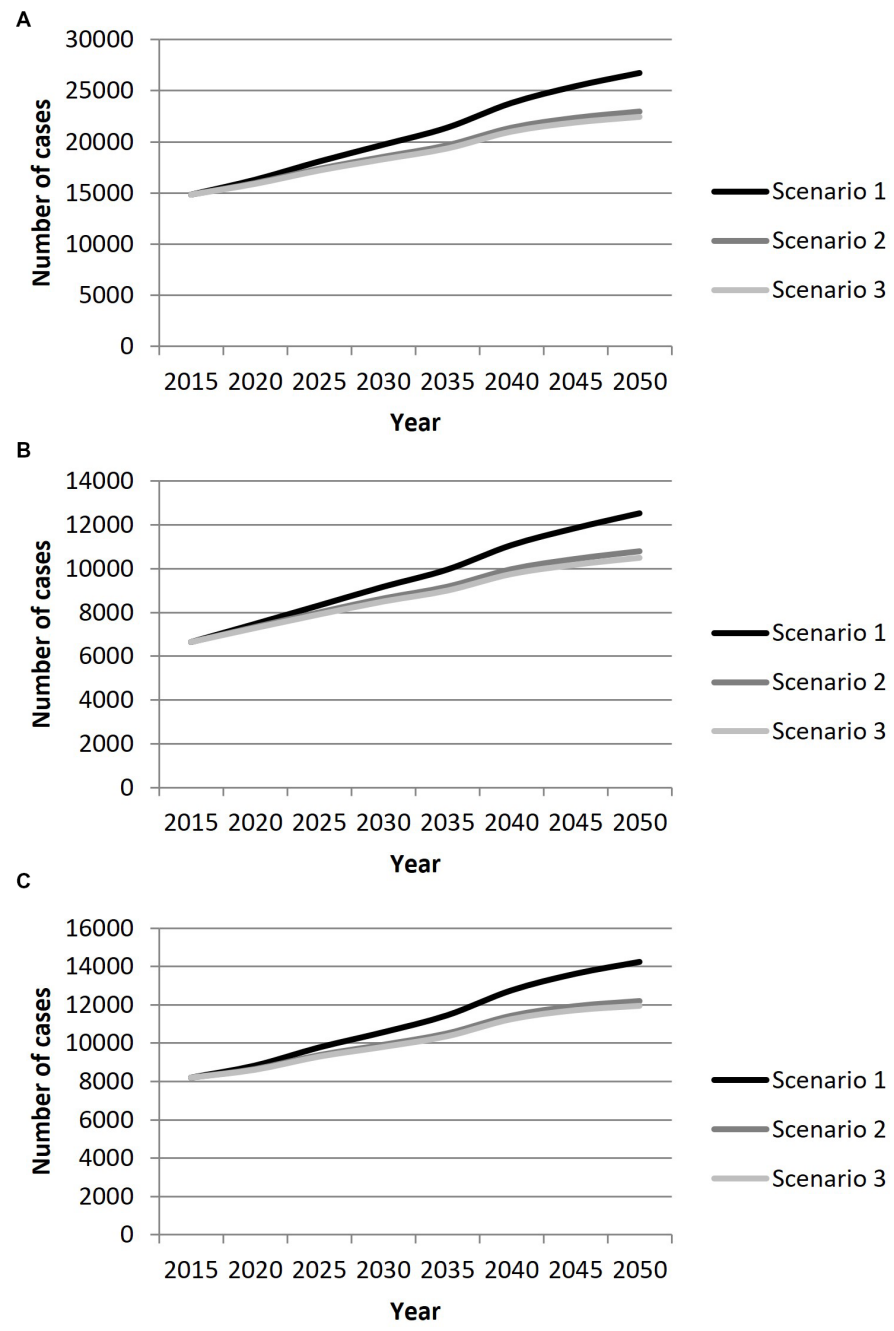
Projection of the number of cases of ischemic stroke with large vessel occlusion in France from 2015 to 2050 in men **(A)** women **(B)** and both **(C)** according to three scenarios: Scenario 1: stable age-and sex-specific incidence rates. Scenario 2: decrease in incidence rates of 0.5%/year in people over 65 years old. Scenario 3: decrease in incidence rates of 0.5%/year in overall population.

A steady increase in the number of IS cases with LVO of the anterior circulation is expected in France from 2015 to 2050, whatever the incidence trend scenario considered (Figure 1). Assuming a stable incidence, this number will increase by 81%, corresponding to 26,763 cases in 2050 (95% CI: 12,918 – 52,008), including 14,243 cases in women (95% CI: 7,666 – 25,813) and 12,520 cases in men (95% CI: 5,252 – 26,195). Considering a decrease in incidence by 0.5%/year in people >65 years old, the expected number of cases will rise by 55% with 22,975 cases in 2050 (95% CI: 10,940 – 45,336) including 12,186 cases in women (95% CI: 6,472 – 22,460), and 10,789 in men (95% CI: 4,468 – 22,876). Finally, if the incidence decreases by 0.5%/year in the entire population, the expected number of cases will increase by 51% with 22,457 cases (95% CI: 10,839 – 43,639) including 11,951 cases in women (95% CI: 6,432 – 21,660) and 10,506 cases in men (95% CI: 4,407 – 21,979).

### 3.2. Evolution of the number of cases by age

**Figure 2:**
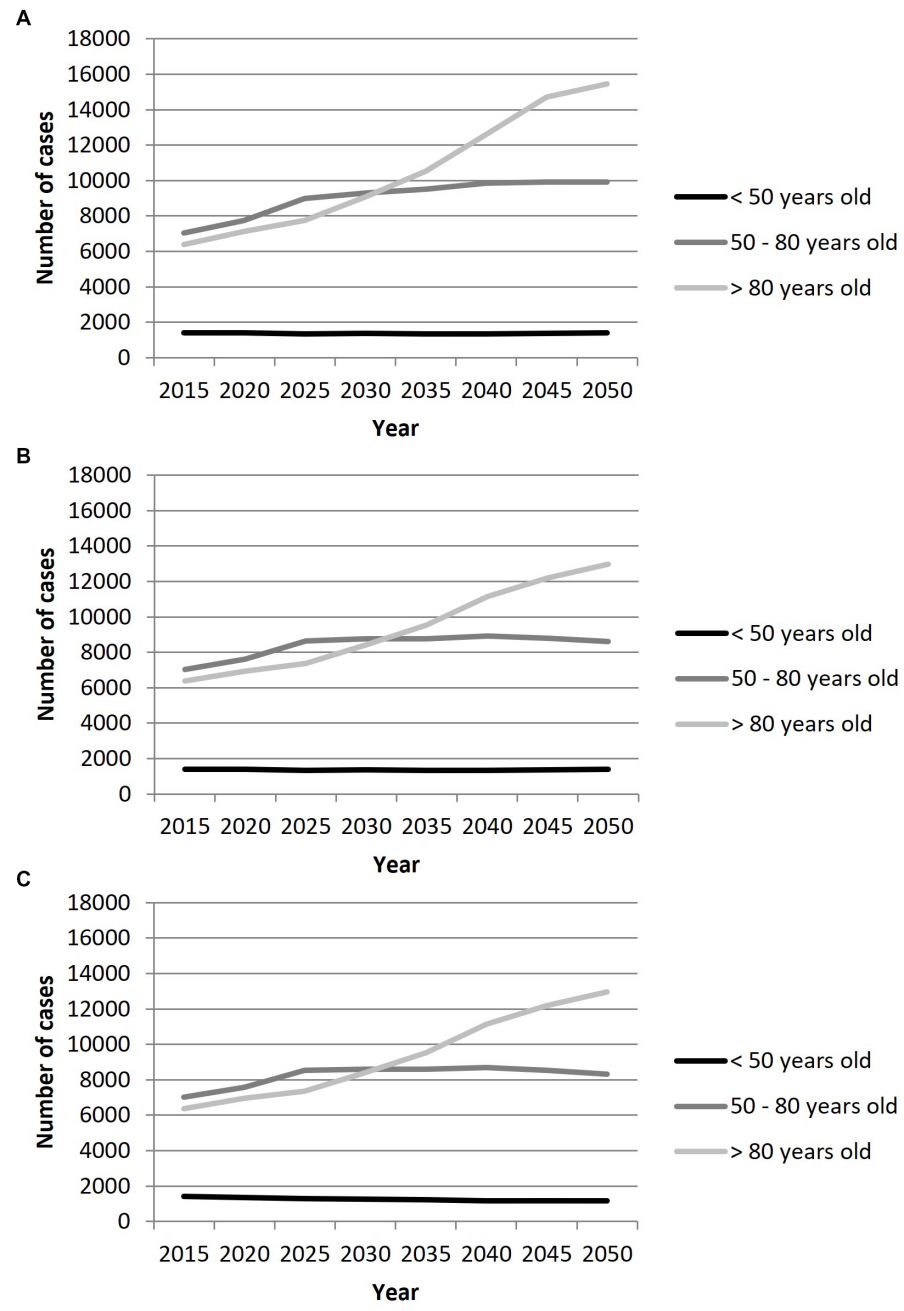
Projection of the number of cases of ischemic stroke with large vessel occlusion in France from 2015 to 2050 by age groups, according to three scenarios: **(A)** Scenario 1 with stable age-and sex-specific incidence rates; **(B)** Scenario 2 with a decrease in incidence rates of 0.5%/year in people over 65 years old; **(C)** Scenario 3 with a decrease in incidence rates of 0.5%/year in overall population.

Due to an expected relative stable population < 50 years old in coming years and considering a stable incidence scenario, the number of cases of IS with LVO will not significantly change (−0.9% between 2015 and 2050) in this age group Figure 2. In case of a decrease in the incidence by 0.5%/year, we expect a decrease of cases by 16.8% in people <50 years old.

Conversely, in the age group 50–80 years old, an increase in the number of cases by 41%, 23 and 18% according to the 3 incidence trends scenario, respectively, will be observed.

Finally, in individuals over 80 years, a steady increase in the number of cases of IS with LVO is expected throughout the study period. The increase is estimated to be by 142% considering a stable incidence scenario, and by 103% if the incidence decreases by 0.5%/year in this age group.

### 3.3. Trends in age distribution of patients with is and LVO

**Figure 3:**
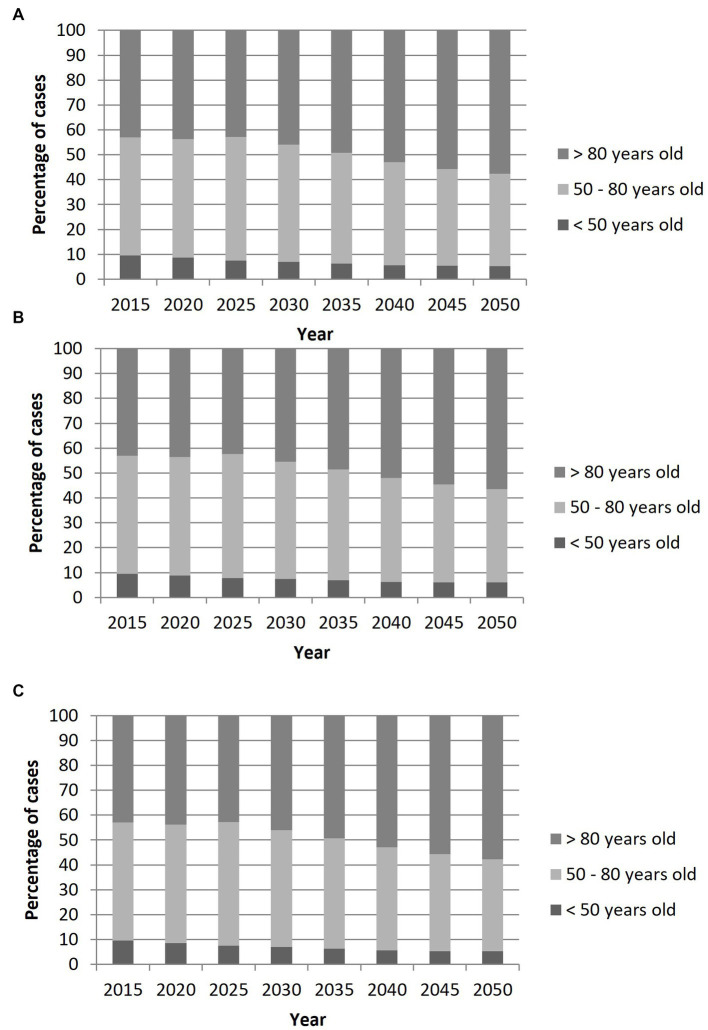
Projection of the age distribution of patients with ischemic stroke with large vessel occlusion in France from 2015 to 2050 according to three scenarios: **(A)** Scenario 1: stable age-and sex-specific incidences rates; **(B)** Scenario 2: decrease in incidence rates of 0.5%/year in people over 65 years old; **(C)** Scenario 3: decrease in incidence rates of 0.5%/year in overall population.

Considering, a stable or a decrease in the incidence in overall population, among IS patients with LVO, the proportion of individuals aged >80 years old will rise from 43% in 2015 to 57.7% in 2050, while that of individuals aged 50–80 years will decrease from 47.5 to 37.1%, and that of individuals <50 years old will decrease from 9.5 to 5.2% Figure 3. When considering a scenario of decreasing incidence by 0.5%/year in people >65 years old, the proportion of patients <50 years old years old will decrease from 9.5 to 6.1%, that of patients aged 50–80 years old will decrease from 47.5 to 37.5%, and that of patients >80 years old will increase from 43.0 to 56.4%.

## 4. Discussion

This study provides original information on nationwide projection of IS with LVO of the anterior circulation in coming years. In the absence of stroke registry covering whole France, these data are of importance to determine future needs for stroke care. Our findings point out a critical increase in the number of cases by 2050 due to the ongoing aging population. This increase will be mainly driven by a rise in the number of IS with LVO in people >80 years.

Our projections were made under the assumption of a stable standardized incidence by 2050, as observed in previous reports of the Dijon Stroke Registry (9). However, divergent findings were noted in other areas. For instance, a decrease by 28% in stroke incidence between the end of the 20th century and the beginning of the 21st century was reported in a meta-analysis including OXVASC study (10) and other European (11–22) and New Zealand (23, 24) population-based registries. Data from the Dijon Stroke Registry were included in this meta-analysis without decrease in the incidence in our population.

Similarly, the Global Burden of Disease (GBD) Study reported a decrease in age-standardised incidence of IS between 1990 and 2019. Therefore, we chose to report additional estimations according to two other scenarios based on an expected decrease in incidence rates (−0.5%/year in people over 65 years old, and −0.5%/year in overall population). Whatever the scenario considered, the projections remained globally unchanged, thus underlying that demographic changes will have a massive impact on the number of IS with LVO, and will overwhelm any improvement in stroke prevention. Of note, we only applied a maximum decrease in incidence of 0.5%/year, which is lower than the decrease reported by Li et al. (10) (−16% over the last 16 years) and projections of Wafa et al. (5) (−10% over 10 years). We chose this option because of specific causes of IS with LVO, i.e., a great frequency of cardioembolic source, especially atrial fibrillation (6). A review of population-and hospital-based studies showed that the recent decline in IS incidence was mostly driven by a decrease in lacunar stroke, whereas cardioembolic IS had increased by 2.4%/year (25), mainly because of a rise in the incidence of atrial fibrillation (26, 27). Therefore, we also proposed a model with no decrease in incidence in the age group under 65 years old, with data showing unchanged incidence in this age group since the beginning of the 21st century (17).

Recently, the Stroke Alliance for Europe anticipated a 34% increase in incident stroke events between 2015 and 2035 in Europe (28), which was consistent with a previous study from the Dijon Stroke Registry in which we estimated that according to demographic projections, the total number of annual stroke cases will increase by 55% between period 2009–2015 and year 2030 (29). Our present study adds to the literature by focusing on nationwide projection of cases of IS with LVO of the anterior circulation, which is of a great importance considering that such patients may be eligible for endovascular therapy, the achievement of which depends on a challenging care organization. Thanks to a systematic review of all arterial imaging exams by stroke-trained investigators, our estimates were based on observed cases of LVO of proximal occlusion, and not derived from proxies such as stroke severity. As others, we recently showed that clinical scales are not relevant enough to predict LVO in IS patients (30). To the best of our knowledge, this is the first time this approach has been used in a population-based study.

In addition, other studies reported the number of mechanical thrombectomy procedures using hospital database to approximate the incidence of IS with LVO (31, 32). Such a method underestimates the true burden of LVO given that it does not consider patients missed for a diagnosis of LVO, or who had no access to this treatment because of stroke care organization failure, or who were excluded from it for medical reasons.

Our results have major implications in both clinical practice and stroke care organization. Indeed, MT is a resource-intensive therapy, with a particular need for interventional neuroradiologists, thus limiting the development of new centers in a context of shortage of specialists. The expected rise in the number of IS with LVO threatens to saturate the existing TM-capable centers and to increase the need for medical transfers of patients. Therefore, the assessment of the number of strokes with proximal occlusion is crucial for the organization of the care pathway and the forecasting of resource needs in order to deploy them in the territories adequately. Then, IS with LVO are generally more severe and are associated with a poorer prognosis despite therapeutic advances, with many sequelae including cognitive or motor disabilities. This is particularly true for older patients, in whom the risk of institutionalization after stroke is high, raising the issue of the need for post-acute care facilities (33). This is all the more true since older patients will account for the vast majority of the increase in the total number of cases (29). It is therefore essential to foresee the weight that these subjects will represent in rehabilitation facilities, long stay units and retirement homes.

In our registry, we noted a stable age-standardized incidence of cerebrovascular disease with an increase in the number of cases in the past years (3). However, these observations need to be balanced. Indeed, we observed in our population of IS with LVO an over-representation of cardio-embolic stroke related to atrial fibrillation (AF) (6), whose prevalence is increasing (26). We cannot exclude that improvements in the diagnosis and treatment of AF may affect the future trends in the incidence of AF-related stroke.

The major strength of our study is the use for the first time of incidence rates obtained from a population-based registry to estimate the future number of cases of IS with LVO. Other studies have used estimations from databases to assess the annual number of MT (31, 32). Conversely, the aim of our study was to estimate the expected number of IS with LVO cases. However, not all these cases result in endovascular management. A study based on UK national registries estimate that approximately one third of IS with LVO are eligible for MT (31, 32). Moreover, the decision of MT takes into account various factors such advancing age, co-morbidities and impaired autonomy. These elements will have to be considered with the increase in the mean age of patients being managed for IS with LVO.

To conclude, knowledge of the future trends in the incidence of IS with LVO is a major issue to accurately anticipate dedicated resources needs. Our study demonstrated that a massive increase in IS with LVO is expected in the coming years, driven by an increasing number of patients.

## Data availability statement

The datasets presented in this article are not readily available because the data that support the findings of this study are available from the corresponding author, upon reasonable request. Requests to access the datasets should be directed to yannick.bejot@chu-dijon.fr.

## Author contributions

GD and YB contributed to the study conception and design. Data collection and analysis were performed by GD and YB. The first draft of the manuscript was written by GD and YB, and both authors commented on previous versions of the manuscript. All authors contributed to the article and approved the submitted version.

## Funding

This work was supported by Santé Publique France, Institut National de la Santé et de la Recherche Médicale (INSERM), and Dijon University Hospital.

## Conflict of interest

YB received honoraria for lectures or consulting fees from BMS, Pfizer, Medtronic, Amgen, Servier, and Novo-Nordisk, outside the submitted work.

The remaining author declares that the research was conducted in the absence of any commercial or financial relationships that could be construed as a potential conflict of interest.

## Publisher’s note

All claims expressed in this article are solely those of the authors and do not necessarily represent those of their affiliated organizations, or those of the publisher, the editors and the reviewers. Any product that may be evaluated in this article, or claim that may be made by its manufacturer, is not guaranteed or endorsed by the publisher.
